# Ionization-induced annealing of pre-existing defects in silicon carbide

**DOI:** 10.1038/ncomms9049

**Published:** 2015-08-12

**Authors:** Yanwen Zhang, Ritesh Sachan, Olli H. Pakarinen, Matthew F. Chisholm, Peng Liu, Haizhou Xue, William J. Weber

**Affiliations:** 1 Materials Science and Technology Division, Oak Ridge National Laboratory, Oak Ridge, Tennessee 37831, USA; 2 Department of Materials Science and Engineering, University of Tennessee, Knoxville, Tennessee 37996, USA; 3 Department of Physics, University of Helsinki, Helsinki FI-00014, Finland; 4 School of Physics, Key Laboratory of Particle Physics and Particle Irradiation (MOE), Shandong University, Jinan 250100, China

## Abstract

A long-standing objective in materials research is to effectively heal fabrication defects or to remove pre-existing or environmentally induced damage in materials. Silicon carbide (SiC) is a fascinating wide-band gap semiconductor for high-temperature, high-power and high-frequency applications. Its high corrosion and radiation resistance makes it a key refractory/structural material with great potential for extremely harsh radiation environments. Here we show that the energy transferred to the electron system of SiC by energetic ions via inelastic ionization can effectively anneal pre-existing defects and restore the structural order. The threshold determined for this recovery process reveals that it can be activated by 750 and 850 keV Si and C self-ions, respectively. The results conveyed here can contribute to SiC-based device fabrication by providing a room-temperature approach to repair atomic lattice structures, and to SiC performance prediction as either a functional material for device applications or a structural material for high-radiation environments.

## Abstract (web summary)

Silicon carbide possesses myriad properties which render it an ideal material for implementation in harsh radiation environments. Here, the authors show that damaged SiC can be repaired through ionization-induced healing, which has broader implications for the design of future radiation-tolerant materials.

## Graphical abstract

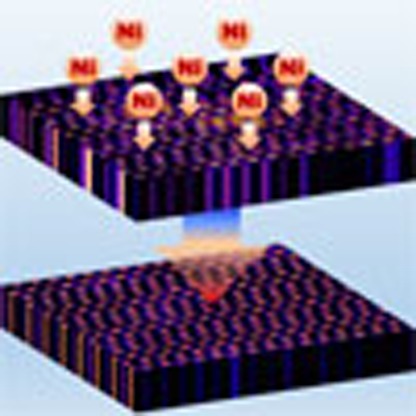

Silicon carbide (SiC) is a wide-band gap semiconductor^1^,^2^,^3^,^4^,^5^, key refractory ceramic^6^,^7^ and radiation-tolerant structural material^8^,^9^,^10^,^11^ that can be functionalized by ion-implantation doping and has great potential for device and structural applications in space and nuclear radiation environments. The capability to retain an ordered atomic structure is essential for reliable device function or material performance in a radiation environment. In these applications, various defects are induced in SiC by interactions with electrons^12^,^13^, ions^14^,^15^,^16^, neutrons^17^ and cosmic rays. During a particle–solid interaction, two distinct energy transfer processes occur: atomic collision cascades and electronic excitation on the atomic and electronic structures, respectively. Often overlooked is the fact that a substantial amount of energy is transferred to electrons directly from such energetic particles or through primary knock-on atoms (PKAs), and this energy can profoundly affect atomic defect evolution. Understanding the effects of electronic energy deposition is particularly important in manufacturing devices, engineering nanoscale structures and predicting material performance for nuclear reactors or space applications where electronic energy deposition and displacement damage occur simultaneously.

In an ordered crystalline structure, the exchange of energy between electrons and atoms, via electron–phonon coupling, leads to local heating. Except for the case of swift heavy ions (see Supplementary Discussion for details), this heating has been either (1) simply neglected in the past under an assumption that the thermal effect is separated from the atomic processes and is dissipated without impact on the atomic structure or (2) more recently considered to enhance defect/damage production (a synergetic effect)^18^, create additional atomic defects (an additive effect)^19^ or cause defect/damage recovery (a competitive effect, induced by swift heavy ion irradiation)^20^. In a defective structure, which is normally the case in many applications, the effects of ionization due to electronic energy loss are largely unknown. Our hypothesis is that ionization effects due to the energy loss to target electrons can anneal pre-existing defects, and therefore may effectively modify or alter microstructure evolution. In the current study, we confirmed this hypothesis and bridged the knowledge gap by quantitatively investigating the ionization effects on pre-existing damage in SiC. A surprisingly low threshold of this recovery process at ∼1.4 keV nm^−1^ induced by MeV ions is determined, which has significant implications for material performance evaluation in extreme radiation environments.

Radiation effects in SiC have been extensively investigated. For ion–solid interactions in SiC at very low energies of up to a few hundred keV (with an electronic energy loss of <1 keV nm^−1^), damage is almost solely attributed to energy transfer to the atomic structure, which results in target atoms being directly displaced from their lattice sites and defects being produced via atomic collision cascades^15^,^21^,^22^. In the high-energy region often referred as swift heavy ions (such as 870 MeV Pb ions with an electronic energy loss of 33 keV nm^−1^), the ion energy is solely deposited to the loosely bound electrons and then, through electron–phonon coupling, transferred into atomic motion. Such ionization processes in materials can either anneal pre-existing damage or induce crystalline-to-amorphous and order-disordered tranformations^20^,^23^,^24^. Most SiC applications utilize ions in the intermediate regime where electronic and nuclear energy losses are both significant^21^,^25^. Examples include: ion implantation, ion beam modification and defect engineering by research institutions and industry; ion beam analytical techniques; and ion simulation to mimic ion and neutron radiation effects in nuclear environments. Limited understanding of the coupled effect on the atomic response of SiC to the two energy deposition pathways is a long-standing roadblock to full utilization of this functional material. Although some defects in SiC can be removed by thermal annealing at temperatures below 1,000 K, with recrystallization occurring at much higher temperatures, such as ∼1,773 K (refs 9, 26), low-temperature manufacturing steps are essential for restoring crystalline order in device applications at nanometre scales.

Previous studies^24^,^27^ have demonstrated that swift heavy ion irradiation with electronic energy deposition ranging from 10 to 33 keV nm^−1^ leads to some damage annealing. Here we report a significant competitive effect, promoted by the electronic energy loss of ions with energies in the intermediate regime accessible to industrial accelerators, whereby nearly complete defect annihilation or damage recovery in pre-damaged 4H-SiC is achievable. The ionization-induced annealing process (recovery of the ordered atomic structure) in SiC has a significant impact on low-temperature processes for eliminating defect production during ion-implantation doping, suppression of single-event upset damage in SiC devices, enhanced radiation tolerance and reliable performance prediction for materials in extreme radiation environments.

## Results

### Displacement damage

**Figure 1:**
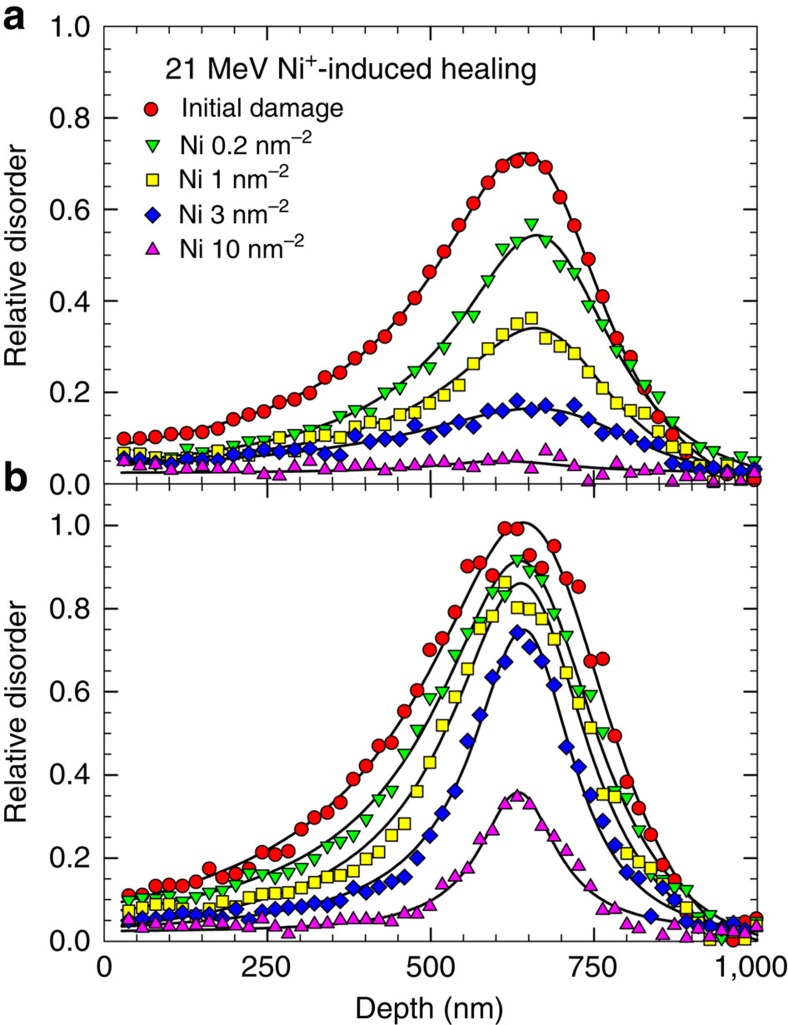
Damage recovery in SiC under 21 MeV Ni^+^ ion irradiations. The initial damage states of (**a**) 0.72 and (**b**) 1.0 were produced by 900 keV Si^+^ with a fluence of 6.3 and 12 ions per nm^2^, respectively. Reduction of relative disorder is clearly evident with the increase of Ni fluence from 0.2 to 10 ions per nm^2^. The combined statistical and measurement uncertainty is represented by the scattered data points from the fitted lines.

To better understand and quantify ionization effects on damage recovery, pre-damaged states were introduced using low-energy ion irradiation with 900 keV Si^+^ self-ions (see Supplementary Methods—Ion Energy Deposition). The high nuclear stopping power (0.38 keV nm^−1^) and comparable electronic to nuclear ratio (1.75), in comparison to other ions (Table 1), are responsible for the displacement damage production. Different fractional disorder levels were produced under different fluences, with peak disorder at a depth of ∼650 nm, *S*_0_, of 0.36, 0.72, and close to 1.0 (the fully amorphous state). Examples are shown in Fig. 1 for the cases of 0.72 and close to 1.0. The sequential evaluation of ionization-induced recovery on these pre-damaged disordered states was carried out at room temperature over a range of electronic energy losses from 1.9 to 7.2 keV nm^−1^ (Table 1). Such irradiation conditions allow a controlled investigation to separately evaluate the ionization effects without introducing significant displacement damage through elastic collisions and to determine the possible threshold of electronic energy loss for the competitive (ionization-induced self-annealing) effects.

### Ionization-induced annealing

Ionization-induced recovery process in SiC (see Supplementary Methods—Ion Energy Deposition) is observed at irradiation conditions as low as 4.5 MeV C, with 21 MeV Ni being the most effective ion beam. Two examples of the annealing effect under 21 MeV Ni^+^ irradiation are shown in Fig. 1. High-disorder profiles of ∼0.72 and 1.00 are produced using 900 keV Si^+^ at fluences of 6.3 and 12 ions per nm^2^, respectively. For 21 MeV Ni the electronic stopping power (*S*_e_) is ∼7 to 8 keV nm^−1^ from the surface to the damage peak region, and the nuclear stopping (*S*_n_) is negligible at levels below ∼0.1 keV nm^−1^, within the first micrometre from the surface (Supplementary Fig. 1). Given the low nuclear stopping values and high *S*_e_/*S*_n_ ratio (Table 1), negligible damage buildup from the nuclear energy deposition within 1 μm of the surface region is expected. This assumption is confirmed as no damage buildup is observed on either the Si or the C sublattice along the <0001> channelling direction in a pristine crystal under 21 MeV Ni^+^ irradiation for ion fluences of up to 10 ions per nm^2^ (Supplementary Fig. 2). Significant damage annealing is observed in the pre-damaged region as a result of sequential Ni irradiations for ion fluences up to 10 ions per nm^2^ (Supplementary Fig. 3). As shown in Fig. 1a,b, clear damage reduction over the entire damage profile is evident after the lowest-fluence irradiation to 0.2 ion per nm^2^. The relative recovery rate depends on the initial disorder level, and a relatively larger annealing effect is observed for the less-damaged sample (*S*_0_=0.72). For this highly disordered sample (Fig. 1a), considerable damage recovery is observed after Ni irradiation at ion fluences up to 3 ions per nm^2^, and the relative disorder decreases from 0.72 to 0.16. At an ion fluence of 10 ions per nm^2^, the Si ion-induced damage is almost fully healed; and the ordered atomic structure is confirmed, as shown by the very low disorder level. For the high-disorder sample with *S*_0_=1.0, a higher-fluence Ni beam is needed to repair the pre-existing damage, as shown in Fig. 1b. Substantial recovery is observed under the Ni irradiations when the ion fluence increases from 3 to 10 ions per nm^2^, with the disorder levels dropping to 0.75 and 0.36, respectively. Additional Ni irradiation is required to fully heal the damaged crystalline structure.

**Figure 2:**
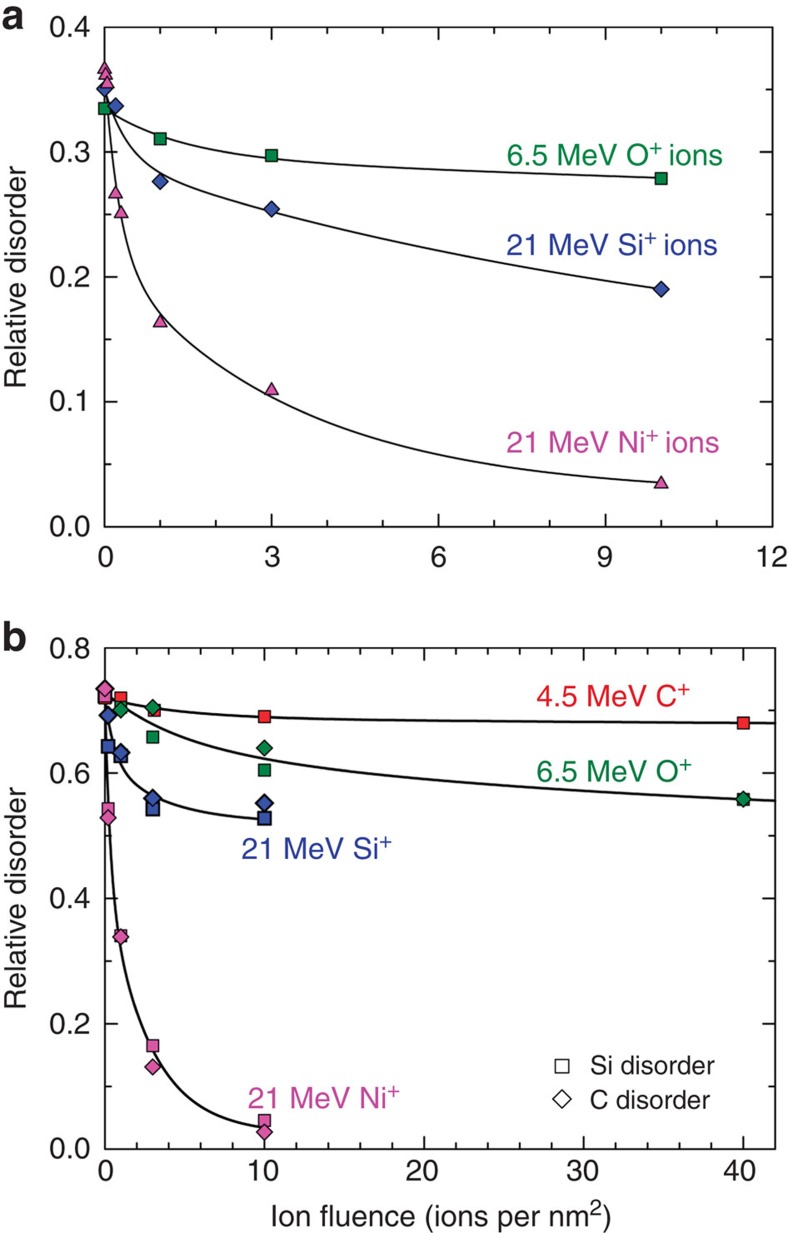
Ion irradiation-induced healing. Two initial relative disorder levels *S*_0_ of (**a**) 0.36 and (**b**) 0.72 are included as representative. While an average disorder determined from both the Si and C sublattices is shown in (**a**), the actual disorder values on the Si and C sublattices are shown in (**b**). The fluence dependence indicates an effective healing power of Ni ions, as compared with Si, O or C ions. The measurement uncertainty is <3% for disorder determined from the Si sublattice and <7% from the C sublattice.

Ionization-induced recovery under MeV C, O, Si and Ni ion irradiation is observed as a reduction in disorder with increasing ion fluence. The damage recovery behaviour in the pre-damaged samples with *S*_0_=0.36 and 0.72 is shown in Fig. 2. The recovery from disorder, averaged from both the Si and the C sublattices, as a function of ion fluence is shown in Fig. 2a for O, Si and Ni irradiation of the *S*_0_=0.36 samples. To demonstrate consistent ionization-induced recovery on both the Si and C sublattices, the disorder recovery determined from both sublattices is presented in Fig. 2b for the *S*_0_=0.72 samples. Compared with C, O and Si irradiation, a significant recovery from Ni ion irradiation is evident in Fig. 2b. In the intermediate MeV energy regime with a high ratio of electronic to nuclear stopping powers (Table 1), the results suggest that energy deposited to the target electronic system can effectively anneal irradiation damage. In addition, the ionization-induced annealing increases with ion mass and ion energy. Moreover, compared with the results from the ions with lower healing power (O and Si) in Fig. 2a,b, the relative recovery (when normalized to the pre-existing disorder level) is nearly a factor of two higher for the samples with the lower initial disorder. At lower levels of disorder, there is an increasing fraction of simple defects, while at higher levels of disorder, more thermally stable defects (for example, clusters and small amorphous domains) are present. Owing to simpler defect types and structure at lower damage levels, recovery is easier in the samples with lower initial disorder.

**Figure 3:**
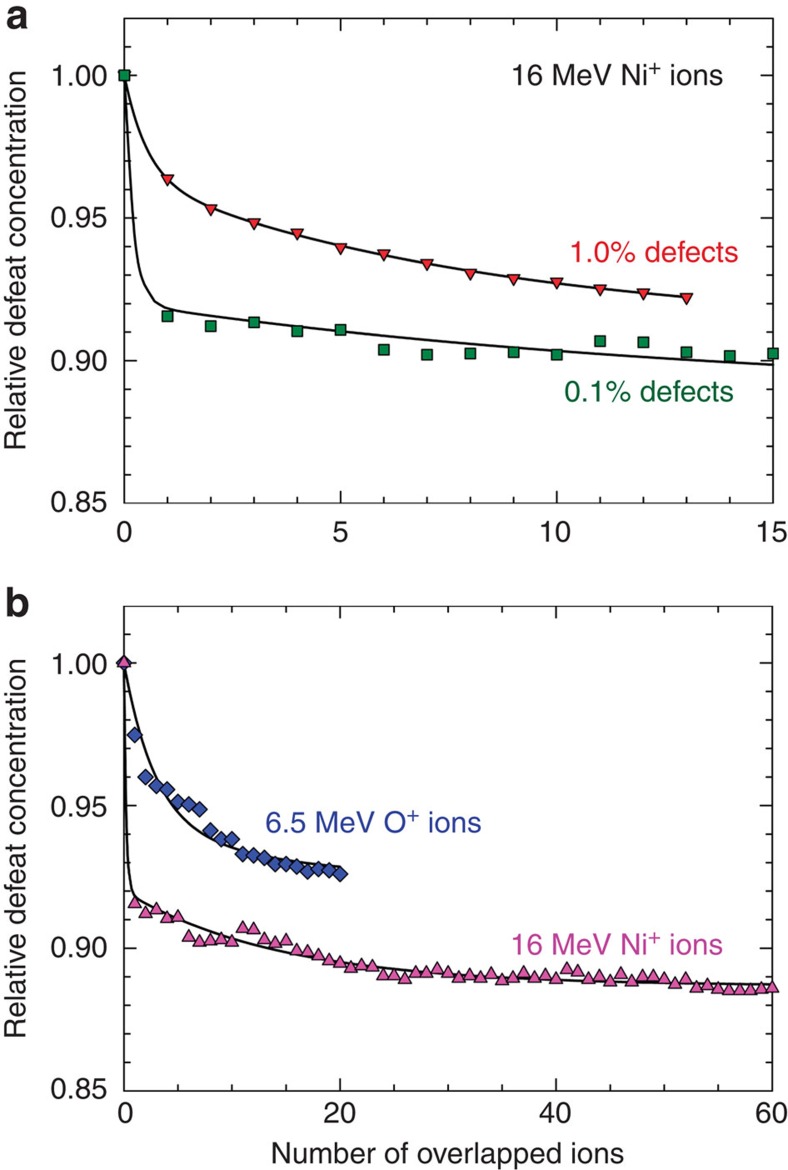
Molecular dynamics simulation of ionization-induced healing. Decrease of the relative defect concentration is shown as a function of overlap ions: (**a**) 16 MeV Ni ions in a simulation cell with initial disorder levels of 1.0 and 0.1% and (**b**) 6.5 MeV O ions and 16 MeV Ni ions in a simulation cell containing 0.1 % Frenkel pairs, respectively. The initial damage state is marked as (0) before the first (1) ion. The statistical error is represented by the scattered data points from the fitted lines.

Under the MeV irradiation in this study, electronic energy deposition (electronic excitation and ionization) is dominant over atomic energy deposition in the pre-damaged region. Electronic excitation (electronic energy transfer), atomic displacement (momentum transfer) and electron–phonon coupling are entangled with equilibrium heating and non-equilibrium excitation processes^21^. To understand the ionization-induced recovery process at the levels of atoms and electrons, a molecular dynamics approach with a thermal spike model is used. Irradiation-induced recovery from the thermal spike (Supplementary Fig. 4) due to 16 MeV Ni ions with an electronic energy loss of ∼7.2 keV nm^−1^ was calculated using cubic 24 × 24 × 24 nm^3^ simulation cells (1.3 million atoms of SiC) containing Frenkel defect concentrations of ∼0.1 and 1.0 % (Fig. 3a). The initial damage is shown as ‘0' overlapping ions. Energetic ions lose their energy as they travel through solids. The Ni energy of 16 MeV was chosen, since this is the average energy of the 21 MeV Ni ions at the depth of the pre-damage peak, ∼650 nm below the sample surface. As shown in Fig. 3, a more effective healing effect is observed for the case with a lower defect concentration, consistent with our experimental results shown in Fig. 1. To evaluate the efficiency of the annealing power with different electronic stopping powers, molecular dynamics simulations of O ion impacts were also performed. The ion energy of 6.5 MeV is chosen for its flat energy deposition profile within the first micrometre of depth in SiC (Supplementary Fig. 1). A comparison of the annealing powers resulting from the thermal spike induced by 6.5 MeV O and 16 MeV Ni ions on an initial defect concentration of 0.1% is shown in Fig. 3b. Significant recovery attributed to the initial ion impact, which heals the most unstable defects, is observed in both cases, similar to what is shown experimentally in Fig. 2. Furthermore, our molecular dynamics simulations suggest that, on average, about half of the healing (decrease of the number of coordination defects in the cell) from one ion is completed within 4 ps from the ion passage, and a few percent stay until 80–100 ps. For the initial ion, this annealing process is even faster, 50% within 1 ps, almost 90 % in 10 ps and ∼1 % occurring during the 80–100 ps time frame. A previous molecular dynamics study^28^ using the same interatomic potential has shown that recovery of close Frenkel pairs occurs at activation energies as low as 0.22 eV for several interstitial configurations on sub-picosecond timescales at 2,000 K (supplementary Fig. 4). Similar molecular dynamics studies^29^ of interstitial self-diffusion in SiC have shown that the transition time for single interstitial hops to be on the order of 0.01 ps at 1,500 K. Moreover, for the same number of overlapping ions, high-energy Ni ions display a much more effective recovery process, again consistent with the experimental results shown in Fig. 2. It is worth noting that both the simulation and the experimental results suggest damage recovery with an exponential dependence on ion fluence or number of overlapping ions.

### Atomic-level microstructure characterization

**Figure 4:**
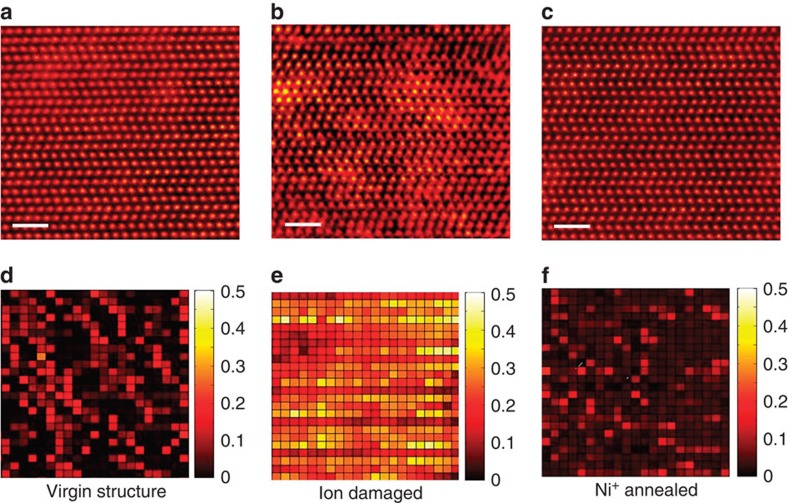
Atomic-level damage recovery. HAADF images (**a**–**c**) and the atomic displacement map (**d**–**f**) of a virgin (**a**) and pre-damaged sample (*S*_0_=0.72) before (**b**,**e**) and after (**c**,**f**) 21 MeV Ni^+^ irradiation to 10 ions per nm^2^. The scale bars on the HAADF images correspond to 1 nm. The vector modulus (**d**–**f**) of the corresponding HAADF images (**a**–**c**) represents the displacement of each atom from its ideal position. The colour bar represents the vector modulus with a measurement error bar of 0.1 Å.

To validate the annealing effect and confirm defect annihilation, high-resolution microstructural analysis was carried out. The top panel of Fig. 4 shows high-angle annular dark field (HAADF) images of the 4H-SiC samples, including a virgin undamaged sample and a pre-damaged sample with *S*_0_=0.72 before and after 21 MeV Ni^+^ irradiation to an ion fluence of 10 ions per nm^2^. The images were all taken at the same depth (∼500 nm below surface) with the sample oriented along the 
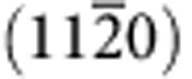
 zone axis. Compared with the undamaged structure (Fig. 4a), larger variation in the atomic contrast is evident in the Si^+^-irradiated SiC (Fig. 4b) as a result of atomic displacement from the irradiation. Surprisingly, the damaged structure is nearly completely healed as a result of irradiation with 21 MeV Ni ions (Fig. 4c). Along with atomic number, the contrast in the HAADF images also contains information regarding lattice distortion resulting from atomic displacements (see Supplementary Methods—Determination of Atomic Displacements, and Supplementary Fig. 5). The atomic displacements from an ideal position of the Si sublattice in SiC are mapped in Fig. 4 (lower panel), which gives a fingerprint of the Si sublattice distortion due to various ion irradiation events corresponding to each atom shown in the top panel. The colour maps in the lower panel represent the vector modulus of the Si atom displacement (Supplementary Fig. 5). Whereas a high level of contrast variation (large atomic displacement from the ideal lattice position) is evident in the 900 keV Si^+^ pre-damaged SiC (Fig. 4e), nearly identical contrast levels are observed in the virgin structure (Fig. 4d) and the one annealed by Ni ion irradiation (Fig. 4f). On the basis of the HAADF images and the detailed atomic displacement analysis, it is quite clear that displacement cascade damage introduced by 900 keV Si^+^ ion irradiation is healed by the electronic energy deposition from the 21 MeV Ni irradiation.

**Figure 5:**
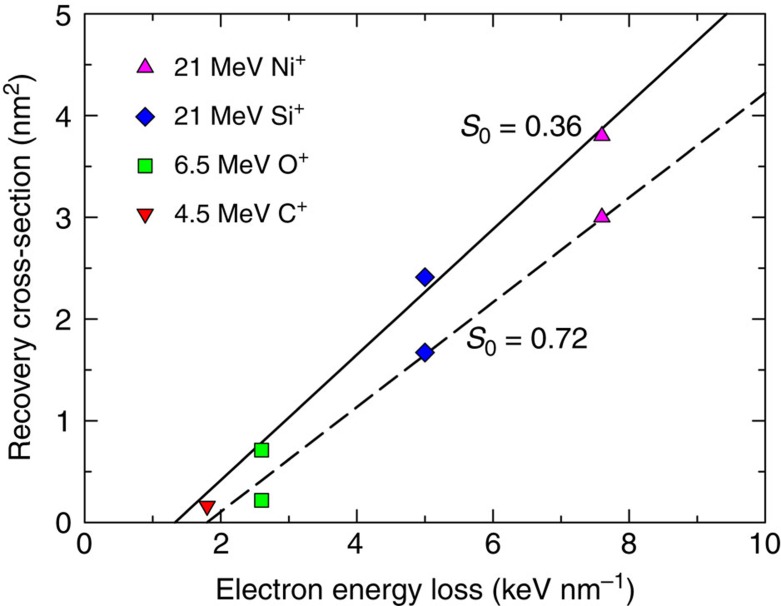
Ionization-induced recovery cross-section. The cross-section is derived from samples with initial disorder levels *S*_0_ of 0.36 and 0.72 as a function of electronic energy loss. The effective cross-section depends on the ions (C, O, Si and Ni) and the initial damage state. The combined measurement and fitting uncertainty is <10%.

To quantify the annealing power, irradiation-induced recovery cross-sections, depending on both the initial disorder level and electronic energy loss, are determined (see Supplementary Methods—Determination of Scattering Cross Section). The results in Fig. 5 show the recovery cross-sections for various ions (C, O, Si and Ni) with an initial relative disorder of 0.72 or 0.36 as a function of electronic energy loss. Significantly increased recovery is observed with increasing electronic energy loss from 1.9 to 7.2 keV nm^−1^ for ions from C to Ni (Table 1) that is attributed to relatively larger recovery cross-sections of 21 MeV Ni compared with those of 4.5 MeV C. For the lower mass of O and C ions, the maximum electronic stopping power (Supplementary Fig. 1) is at ∼2.6 (6.5 MeV O) and 1.9 keV nm^−1^ (4.5 MeV C); the effective recovery cross-sections are, therefore, limited. The experimental and modelling results (Figs 1, 3 and 5) also indicate that the recovery process depends on the pre-existing defect structure and is more effective at lower initial damage levels or defect concentrations. The effective cross-section, therefore, depends on the initial damage state, which cannot simply be described based solely on the electronic stopping power. A threshold at about 1.4 keV nm^−1^ is predicted based on a linear fit of the low-disorder state (0.36).

## Discussion

In our combined approach based on ion channelling measurements, atomic-level microstructural analysis and large-scale atomistic simulations, we quantify the effects of electronic energy loss on pre-irradiation-induced lattice damage in SiC. These results provide a scientific understanding of the effects of ionization on ceramics damaged by irradiation. Such electron excitation-induced material modifications should be taken into account for *in situ* transmission electron microscope studies under ion irradiation, in which simultaneous ionization-induced damage recovery/evolution should be considered. Moreover, our results demonstrate that electronic energy loss from ions and their PKAs can repair damaged SiC lattices at unexpectedly low values of electronic energy deposition, with a threshold value of 1.4 keV nm^−1^ at room temperature. The threshold PKA energies required to activate these processes in neutron-irradiation environments at room temperature are, therefore, 750 and 850 keV for Si and C PKAs, respectively. It is known that SiC, as a key nuclear material for extreme radiation environments^30^, is considered for use as a structural material and a fuel coating in fission reactors^9^,^31^, for structural components in fusion reactors^32^ and as an inert matrix for transmutation of plutonium and other radioactive waste^33^,^34^. SiC is also considered for use as an accident-tolerant cladding for light water reactors and in structural components for advanced high-temperature gas-cooled reactors. The energies of PKAs created by fusion neutrons and accelerator-based neutron sources, as well as the energies of ions used to investigate neutron damage in materials, are in the intermediate regime where significant ionization effects demonstrated in the current work should not be overlooked.

The significant ionization-induced annealing power can effectively remove nearly all the radiation-induced defects, which is different from the ion beam-enhanced crystallization process that relies on higher temperature to promote atomic mobility where substantial crystalline damage remains. While previous work has focused on high-energy ions and shown ionization effects from swift heavy ions (W, Pb, Bi, etc.)^24^,^27^,^35^,^36^ with relatively high thresholds (>10 keV nm^−1^), we call attention to non-negligible ionization effects from light ions (C, O, Si and Ni) with energies of a few MeVs. From separate effect experiments and modelling work, ion annealing at room temperature in the low-energy end of the MeV range, with a low threshold of electronic energy deposition, is conclusively demonstrated. It is worth noting that a threshold of >15 keV nm^−1^ has been reported for defect annealing induced by electronic energy loss over a wider range of pure metals^37^. Owing to different bonding and energy dissipation pathways in metal (metallic bonding) and SiC (covalent and ionic bonding in ceramics), the difference in threshold values is largely attributed to significant difference in energy deposition profiles between pure metals and SiC, resulting from much higher electronic and thermal conductivities, as well as larger values of electron mean free path in metals. This recovery process with a low threshold (1.4 keV nm^−1^) may significantly extend the performance lifetime of SiC in fusion reactor environments. In terms of fundamental research, this ionization-induced annealing power may have a significant impact on prediction of radiation damage accumulation in SiC and other nuclear materials responding to fast neutrons, accelerator-based neutrons or surrogate ions. Consideration of ionization-induced recovery in SiC is critical for reliable performance evaluation.

In summary, we have investigated ionization-induced healing of ballistic damage in SiC and identified an unexpectedly low threshold value of electronic energy loss for initiating the healing process at room temperature. Our findings are validated by a substantial reduction in dechannelling yield due to the effective annihilation of a high concentration of interstitials and small defect clusters over a sub-micrometre depth, by evidence of a repaired crystalline structure with much less observed displacements at the atomic level and by insights into corresponding defect dynamic processes revealed by molecular dynamics simulations. Understanding this recovery mechanism in SiC has significant implications for the study of irradiation effects in other ceramics for applications in extreme radiation environments. Scientific advances based on this work not only will facilitate the design of radiation-tolerant materials for advanced nuclear energy systems and space exploration but also will contribute to a foundation for the design and control of material properties. That foundation will enable broad advances in device fabrication, sustainable energy technologies and national security involving materials subjected to ion beam modification or severe radiation environments.

## Methods

### Ion irradiation

Quantitative damage recovery studies were performed by first introducing different disorder levels containing various concentrations of Frenkel pairs and defect clusters in SiC through ballistic collision processes initiated by low-energy 900 keV Si^+^ ions. The damage profile is peaked at ∼650 nm from the surface. Ionization-induced recovery was quantitatively investigated using high-energy ions of 4.5 MeV C, 6.5 MeV O, 21 MeV Si and 21 MeV Ni at room temperature (see Supplementary Methods—Ion Energy Deposition). The ion flux was 1.7 × 10^12^ cm^−2^ s^−1^ for C and O, 1.7 × 10^11^ cm^−2^ s^−1^ for Si and 1.5 × 10^11^ cm^−2^ s^−1^ for Ni, respectively. These MeV ions deposit their energy with very high ratios of electronic to nuclear energy loss (Table 1).

### Ion beam analysis

Irradiation-induced damage in crystalline samples was quantified using backscattering techniques: Rutherford backscattering spectrometry (RBS) and non-Rutherford backscattering spectrometry (NRBS). Helium ions with an energy of 3.5 MeV were employed to significantly enhance the scattering cross-section of C atoms, and the disorder analysis on both Si (RBS) and C (NRBS) sublattices along the <0001> direction was performed from a single channelling backscattering measurement (see Supplementary Methods—Ion Channeling Measurements). If a crystal contains displaced lattice atoms, there will be an increased yield resulting from direct backscattering and dechannelling of the probing ions due to the interaction with the displaced atoms. Following the 900 keV Si^+^ irradiations, subsequent *in situ* channelling measurements were carried out, with a Si detector located at a scattering angle of 155° relative to the incoming beam, before and after the additional MeV irradiations. The channelling spectra were analysed using an iterative procedure to achieve the relative disorder level^25^.

### Molecular dynamics

Large-scale classical molecular dynamics studies were performed that simulated electronic energy loss generated by passing ions^38^. The radial distribution of electronic heating from the passing incident ions (supplementary Fig. 4) is determined by applying the thermal spike model^39^. The local temperature from ionization as a function of time and radial distance from the ion trajectory was calculated and transferred to atoms along the ion track as kinetic energy at the beginning of the molecular dynamics simulation (see Supplementary Methods—Molecular Dynamics Simulations). Irradiation-induced recovery due to a series of directly overlapping 16 MeV Ni ions, each separated by a 120-ps relaxation to 300 K temperature, was calculated using simulation cells containing Frenkel defects of both ∼0.1 and 1.0 %, as shown in Fig. 3a. The 16-MeV Ni and 6.5-MeV O results were compared using a cell with an initial disorder level of 0.1%.

### STEM characterization

Samples were prepared for scanning transmission electron microscope (STEM) analysis using a focused ion beam (FIB) in a cross beam Zeiss Auriga FIB/SEM. A lamella of ∼800-nm thickness was prepared using a gallium FIB beam probe with a current of 2 nA at 30 KV. The current was gradually reduced to 10 pA to minimize FIB beam damage while thinning the lamella to ∼100 nm. As a final step, a Fischione 1040 nanomill was used to further thin the sample at 900 eV from each side of the sample at an angle of ±7° for 5 min. HAADF imaging was performed on various samples in a fifth-order aberration-corrected STEM (Nion UltraSTEM200) operating at 200 KV. A detector with an inner angle of 65 mrad was used to collect electrons for HAADF imaging. The electron probe current and the exposure time/pixel for imaging in the experiment were 30 pA and 16 μs, respectively, to minimize the electron beam-induced modification.

## Additional information

**How to cite this article:** Zhang, Y. *et al.* Ionization-induced annealing of pre-existing defects in silicon carbide. *Nat. Commun.* 6:8049 doi: 10.1038/ncomms9049 (2015).

## Supplementary Material

Supplementary Information Supplementary figures 1-5, Supplementary Discussion, Supplementary Methods and Supplementary References

## Figures and Tables

**Table 1:** Ion irradiation condition and predicted stopping powers using the stopping and range of ions in matter (SRIM) code.

**Ions**	**Energy**	**d*****E*****/d*****x***_**ele**_**-s**	**d*****E*****/d*****x***_**Nucl**_**-s**	**Ratio-s**	**d*****E*****/d*****x***_**ele**_**-p**	**d*****E*****/d*****x***_**Nucl**_**-p**	**Ratio-p**
Ni	21	8.2	6.6 × 10^−2^	124	7.6	7.9 × 10^−2^	96
Si	21	5.0	1.0 × 10^−2^	483	5.0	1.2 × 10^−2^	422
O	6.5	2.6	6.0 × 10^−3^	431	2.6	7.8 × 10^−3^	334
C	4.5	1.8	3.7 × 10^−3^	496	1.8	4.7 × 10^−3^	383
Si	0.9	1.6	1.2 × 10^−1^	13	0.65	3.8 × 10^−1^	1.75

Medium mass ions, their energy (MeV), electronic stopping powers d*E*/d*x*_ele_ (keV nm^−1^) and nuclear stopping powers d*E*/d*x*_Nucl_ (keV nm^−1^) at both sample surface (-s) and 650 nm, where the damage peak (-p) is produced from the 900 keV Si^+^ irradiation are summarized. The corresponding ratio of d*E*/d*x*_ele_ to d*E*/d*x*_Nucl_ is also calculated at the surface and the damage peak.

## References

[b1] Madar R. Materials science: silicon carbide in contention. Nature 430, 974–975 (2004) . 15329702 10.1038/430974a

[b2] Lee T-H., Bhunia S. & Mehregany M. Electromechanical computing at 500°C with silicon carbide. Science 10, 1316–1318 (2010) . 20829479 10.1126/science.1192511

[b3] Obermayer D., Gutmann B. & Kappe C. O. Microwave chemistry in silicon carbide reaction vials: separating thermal from nonthermal effects. Angew. Chem. Int. Ed. 48, 8321–8324 (2009) . 10.1002/anie.200904185 19784993

[b4] Eddy C. R. Jr. & Gaskill D. K. Silicon carbide as a platform for power electronics. Science 324, 1398–1400 (2009) . 19520947 10.1126/science.1168704

[b5] Nakamura D. *et al.* Ultrahigh-quality silicon carbide single crystals. Nature 430, 1009–1012 (2004) . 15329716 10.1038/nature02810

[b6] Tredway W. K. Toughened ceramics. Science 282, 1275–1275 (1998) .

[b7] Ishikawa T. *et al.* A tough, thermally conductive silicon carbide composite with high strength up to 1600°C in air. Science 282, 1295–1297 (1998) . 9812889 10.1126/science.282.5392.1295

[b8] Ochedowski O. *et al.* Graphitic nanostripes in silicon carbide surfaces created by swift heavy ion irradiation. Nat. Commun. 5, 3913 (2014) . 24905053 10.1038/ncomms4913

[b9] Jones R. H. *et al.* Promise and challenges of SiCf/SiC composites for fusion energy applications. J. Nucl. Mater. 307-311, 1057–1072 (2002) .

[b10] Snead L. L. *et al.* Handbook of SiC properties for fuel performance modeling. J. Nucl. Mater. 371, 329–377 (2007) .

[b11] Zhang Y. *et al.* Nanoscale engineering of radiation tolerant silicon carbide. Phys. Chem. Chem. Phys. 14, 13429–13436 (2012) . 22948711 10.1039/c2cp42342a

[b12] Ishimaru M., Zhang Y., Shannon S. & Weber W. J. Origin of radiation tolerance in 3C-SiC with nanolayered planar defects. Appl. Phys. Lett. 103, 033104 (2013) .

[b13] Inui H., Mori H., Suzuki T. & Fujita H. Electron-irradiation-induced crystalline-to-amorphous transition in *β*-SiC single crystals. Phil. Mag. B 65, 1–14 (1992) .

[b14] Jin K. *et al.* Electronic stopping powers for heavy ions in SiC and SiO_2_. J. Appl. Phys. 115, 044903 (2014) .

[b15] Zhang Y., Gao F., Jiang W., McCready D. E. & Weber W. J. Damage accumulation and defect relaxation in 4H-SiC. Phys. Rev. B 70, 125203 (2004) .

[b16] Jiang W. *et al.* Response of nanocrystalline 3C silicon carbide to heavy-ion irradiation. Phys. Rev. B 80, 161301(R) (2009) .

[b17] Snead L. L. & Hay J. C. Neutron irradiation induced amorphization of silicon carbide. J. Nucl. Mater. 273, 213–220 (1999) .

[b18] Toulemonde M. *et al.* Synergy of nuclear and electronic energy losses in ion-irradiation processes: the case of vitreous silicon dioxide. Phys. Rev. B 83, 054106 (2011) .

[b19] Zhang Y. *et al.* The effect of electronic energy loss on irradiation-induced grain growth in nanocrystalline oxides. Phys. Chem. Chem. Phys. 16, 8051–8059 (2014) . 24651953 10.1039/c4cp00392f

[b20] Debelle A. *et al.* Combined experimental and computational study of the recrystallization process induced by electronic interactions of swift heavy ions with silicon carbide crystals. Phys. Rev. B 86, 100102 (2012) .

[b21] Zhang Y., Weber W. J., Jiang W., Hallén A. & Possnert G. Damage evolution and recovery on both Si and C sublattices in Al-implanted 4H-SiC studied by RBS and NRA. J. Appl. Phys. 91, 6388 (2002) .

[b22] Weber W. J., Duffy D. M., Thomé L. & Zhang Y. The role of electronic energy loss in ion beam modification of materials. Curr. Opin. Solid State Mater. Sci. 19, 1–11 (2015) .

[b23] Benyagoub A. & Audren A. Mechanism of the swift heavy ion induced epitaxial recrystallization in predamaged silicon carbide. J. Appl. Phys. 106, 083516 (2009) .

[b24] Benyagoub A., Audren A., Thomé L. & Garrido F. Athermal crystallization induced by electronic excitations in ion-irradiated silicon carbide. Appl. Phys. Lett. 89, 241914 (2006) .

[b25] Zhang Y., Debelle A., Boulle A., Kluth P. & Tuomisto F. Advanced techniques for characterization of ion beam modified materials. Curr. Opin. Solid State Mater. Sci. 19, 19–28 (2015) .

[b26] Schmidt H. *et al.* Crystallization kinetics of amorphous SiC films: influence of substrate. Appl. Surf. Sci. 252, 1460–1470 (2005) .

[b27] Thomé L. *et al.* Combined effects of nuclear and electronic energy losses in solids irradiated with dual-ion beam. Appl. Phys. Lett. 102, 141906 (2013) .

[b28] Gao F. & Weber W. J. Recovery of close Frenkel pairs produced by low energy recoils in SiC. J. Appl. Phys. 94, 4348 (2003) .

[b29] Gao F. *et al.* Atomistic study of intrinsic defect migration in 3C-SiC. Phys. Rev. B 69, 245205 (2004) .

[b30] Snead L. L. *et al.* Stability of SiC-matrix microencapsulated fuel constituents at relevant LWR conditions. J. Nucl. Mater. 448, 389–398 (2014) .

[b31] Xiao H. *et al.* Near-surface and bulk behavior of Ag in SiC. J. Nucl. Mater. 420, 123–130 (2012) .

[b32] Zinkle S. J. & Busby J. T. Structural materials for fission and fusion energy. Mater. Today 12, 12–19 (2009) .

[b33] Verrall R. A., Vlajic M. D. & Krstic V. D. Silicon carbide as an inert-matrix for a thermal reactor fuel. J. Nucl. Mater. 274, 54–60 (1999) .

[b34] Somiya S. ed. Handbook of Advanced Ceramics: Materials, Applications, Processing, and Properties 2nd edn Elsevier (2013) .

[b35] Zinkle S. J., Skuratov V. A. & Hoelzer D. T. On the conflicting roles of ionizing radiation in ceramics. Nucl. Instrum. Methods B 191, 758–766 (2002) .

[b36] Itoh N., Duffy D. M., Khakshouri S. & Stoneham A. M. Making tracks: electronic excitation roles in forming swift heavy ion tracks. J. Phys. Cond. Matter 21, 474205 (2009) . 10.1088/0953-8984/21/47/474205 21832484

[b37] Wang Z. G. *et al.* Defect production and annealing induced by electronic energy loss in pure metal. Nucl. Instrum. Methods B 135, 265–269 (1998) .

[b38] Backman M. *et al.* Molecular dynamics simulations of swift heavy ion induced defect recovery in SiC. Comput. Mater. Sci. 67, 261 (2013) .

[b39] Toulemonde M. *et al.* Experimental phenomena and thermal spike model description of ion tracks in amorphisable inorganic insulators. Mat. Fys. Medd. Dan. Vid. Selsk 52, 263 (2006) .

