# Supplementary material for: Ionization-induced annealing of pre-existing defects in silicon carbide
Source: Nat Commun. 2015 Aug 12;6:8049. doi: 10.1038/ncomms9049 (PMC4557342; doi:10.1038/ncomms9049)
Supplement: Supplementary Information — Supplementary figures 1-5, Supplementary Discussion, Supplementary Methods and Supplementary References [file ncomms9049-s1.pdf]

Supplementary Figures

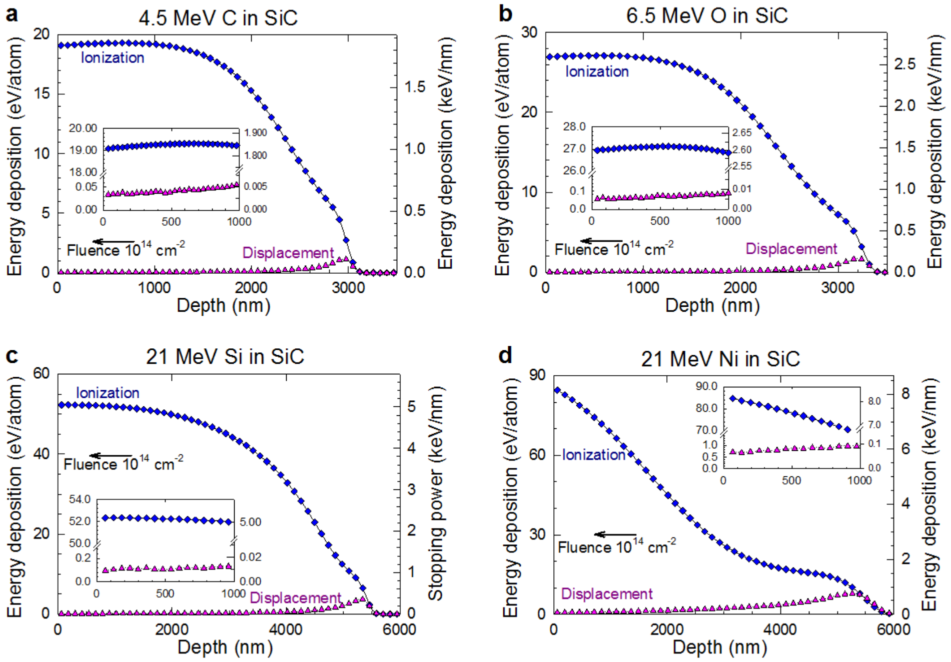

Supplementary Figure 1. SRIM-predicted stopping powers. Electronic and nuclear (left axis) energy deposition (eV atom^{-1}) by (**a**) 4.5 MeV C, (**b**) 6.5 MeV O, (**c**) 21 MeV Si, and (**d**) 21 MeV Ni irradiations to a fluence of 1 ion nm^{-2} in SiC. Also included on the right axis are the electronic and nuclear stopping powers (keV nm^{-1}) under the corresponding irradiation conditions. Details of the SRIM predictions in the first micrometers of the surface are shown as an inset.

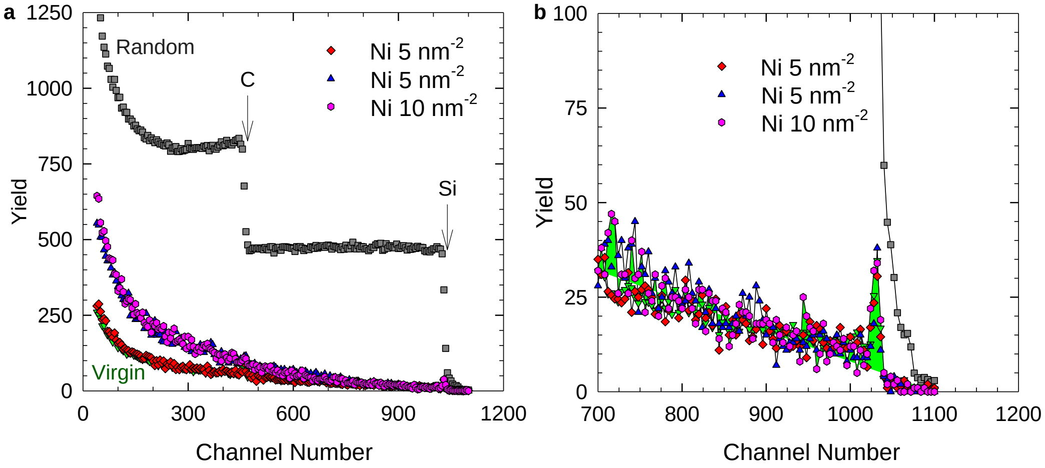

Supplementary Figure 2. Ion channeling characterization of high-energy ion damage. Comparison of the channeling spectra along the <0001> direction in SiC under 21 MeV Ni irradiation with ion fluences of 1, 5, and 10 ions nm^{-2} is shown in (**a**), with detailed comparison of backscattering yield on the Si sublattice in (**b**). Channeling and random spectra from an undamaged SiC sample are also included as references to evaluate the intrinsic crystalline quality and pure Ni irradiation-induced damage in the crystalline structure. The combined statistical and measurement uncertainty is less than 5%.

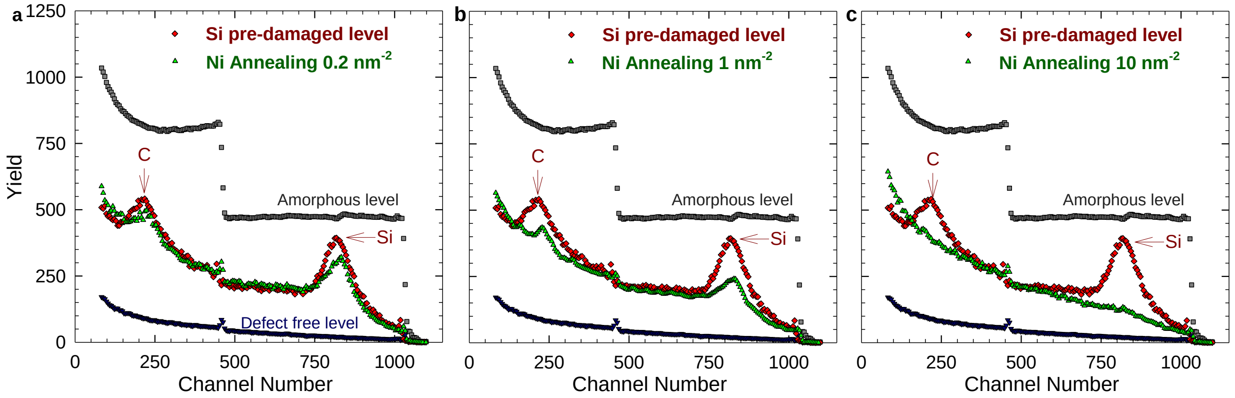

Supplementary Figure 3. Ionization-induced damage annealing. Comparisons of the RBS spectra along the <0001> direction in SiC in the pre-damaged sample before and after ion annealing with 21 MeV Ni ions to ion fluence of (**a**) 0.2 nm^{-2}, (**b**) 1 nm^{-2}, and (**c**)10 nm^{-2}. The virgin and random spectra are provided as references. The pre-damage region was produced using 900 keV Si ions to an ion fluence of 6.3 ions nm^{-2}. The initial damage state and the reduction in backscattering yield due to Ni-induced defect annihilation on both the Si and C sublattices are evident. The measurement uncertainty is represented by the scattered data points.

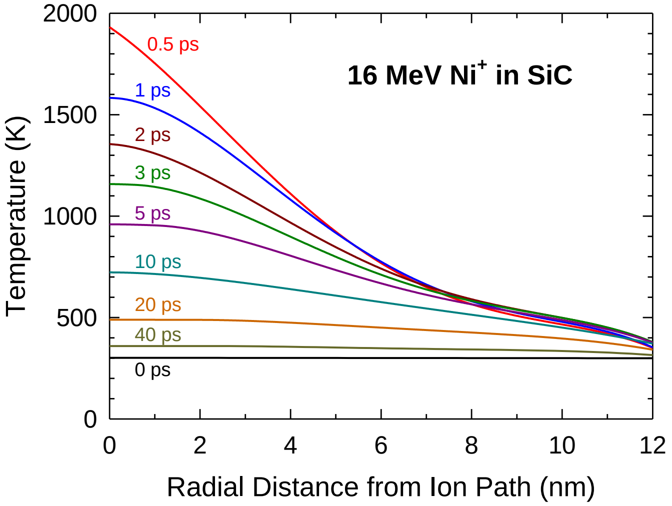

Supplementary Figure 4. Ionization-induced thermal spike. The radial and temporal evolution of the atomic temperature from the two temperature model for 16 MeV Ni ions in the pre-damaged system containing 0.1 % Frenkel pairs at room temperature.

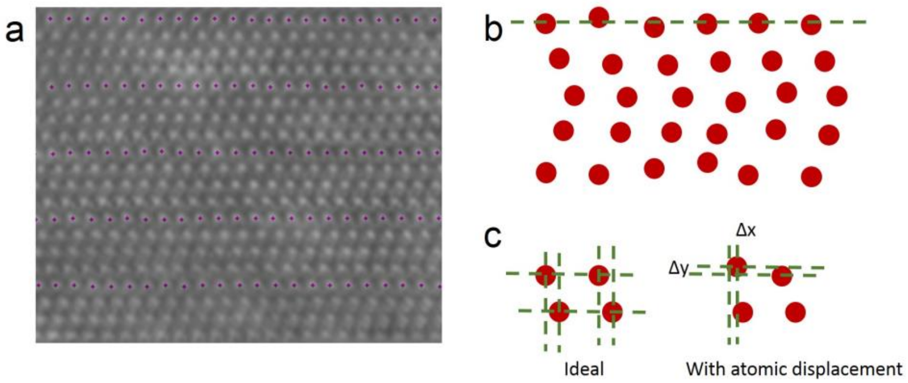

Supplementary Figure 5. Method to determine atomic displacements. (**a**) A representative HAADF image is used to describe the measurement of atomic displacement. The center of each atom position is determined and is indicated by a pink marker in the image. (**b**) Model showing 4H-SiC atomic arrangement in which atoms are displaced from their original positions. The green trend line is drawn as a reference for the displacement with regard to the first atom of the row. (**c**) The displacement measurement (Δx and Δy) from the ideal location of each atom. The measurement error bar is of 0.1Å.

Supplementary Discussion

Ionization Effects and Ionization–induced Recovery Processes in SiC

Silicon carbide (SiC) is a wide band-gap material with broad potential for power electronics, optoelectronics, and electro-mechanical devices that are capable of operating at extreme conditions. It has recently attracted much attention for quantum computing utilizing luminescence of divacancies as a potential qubit[1,2], for applications in nonlinear optics[3], and for use as single-photon source with ultra-brightness at room temperature[4]. Based on coupling of its superior mechanical strength and semiconductor properties, SiC is considered as exceptionally suitable for electronic device applications in space exploration, where exposure to extreme temperatures and cosmic radiation (including ionization radiation) can cause device failure. Understanding of defect production, irradiation damage, and annealing processes is the scientific foundation that underpins the survivability of advanced electronics to single-ion event upsets and is critical to predict the performance of sub-100 nm electronics. In addition to its being a key nuclear material for extreme radiation environments[5-9], potential benefits of SiC cladding for accident-tolerant nuclear fuel systems, including increased corrosion resistance and reduced oxidation and hydrogen generation under steam attack (off-normal conditions) are highlighted by the Light Water Reactor Sustainability Program[10]. A common aspect in many of these applications is that SiC may be subjected to changes in critical properties under the far-from-equilibrium conditions induced by energy deposition from electrons, ions, neutrons, or cosmic rays, all of which have the potential to introduce defects and evolve nanostructures. This is a critical problem, as these defects may deteriorate device performance and material functionality, and can become stepping stones for catastrophic events, such as device failure in space missions or loss of structural integrity in nuclear reactors.

Energy deposition, electronic excitation and energy/heat dissipation are subjects of importance to many fields, including applications in nuclear energy and the semiconductor industry. It is worth noting that the primary knock-on atoms created by fast neutrons, as well as the ions used to investigate neutron damage in nuclear materials or introduce dopants for device fabrication (as thermal diffusion efficiency is very limited at low-temperature in SiC), are intermediate-energy ions that transfer substantial energy to both the electronic and atomic structures[11,12]. The response of materials to such energy deposition depends on complex interactions, including electronic excitations, electron-electron scattering, electron-phonon

coupling, electronic and atomic defect formation, all of which affect the microstructural evolution. In recent years, the connection between electronic energy loss mechanisms and atomic processes are described by a thermal spike model, in which the electrons in the vicinity of the ion path undergo a large degree of excitation due to inelastic energy transfer from the ion, electron-electron scattering, subsequently transfer energy via electron-phonon coupling to atoms in the same region, and eventually causing local heating. The energy dissipation within the electronic structure and transfer to the atomic structure is described as an inelastic, or ionization-induced, thermal spike, which is used as the input for the MD simulations. In most cascade simulations, the conventional assumption is that the energy deposited into the electronic subsystem will ultimately be dissipated by non-damage (or non-recovery) mechanisms and that local heating from electron excitation has no significant impact on defect production. Therefore, the inelastic energy deposition is taken out of the system. This approach assumes that the energy deposited to the electronic and atomic subsystems are independent and non-correlated. A two-temperature model[13,14] describes how the energy deposited to electrons is dissipated in the electronic structure and eventually transferred to atomic nuclei as an ionization-induced thermal spike. This two-temperature model provides a better physically-based description over the conventional approaches that do not consider electron excitation and the corresponding heat dissipation to the atomic structure.

Studies on ionization effects in ceramics have largely focused on swift heavy ions with ion energies from a few hundreds MeV to GeV (an electronic energy loss of a few tens keV nm^{-1}). Because of a lack of in-depth understanding of the ionization effects in SiC, such effects have largely been ignored in the intermediate energy regime (up to a few tens MeV with an electronic energy loss of a few keV nm^{-1}). The present study involves several experimental and modeling techniques to describe ionization effects on defect annealing of irradiation damage in SiC. We show that the energy transferred to the electron system of SiC by energetic ions via inelastic ionization processes can effectively heal pre-existing defects and restore the structural order with an unexpectedly low threshold at ~1.4 keV nm^{-1}, a process that should not be overlooked.

Supplementary Methods

Ion Energy Deposition

Low-energy self-ions (Si) were chosen to produce damage within the first micrometer from the sample surface. High-energy ions were selected to penetrate the pre-damaged layer and deposit substantial energy to target electrons across the pre-damaged region. The damage profile from the low-energy Si irradiation was predicted in displacements per atom (dpa) using the Stopping and Range of Ions in Matter (SRIM) code[15] under full-cascade simulations, and the electronic and nuclear energy losses of the incident high-energy ions as a function of depth were calculated using SRIM under a quick calculation of damage. The assumed sample density in the calculations was the theoretical density of 3.21 g cm^{-3}, with threshold displacement energies of 20 and 35 eV for the C and Si sublattices, respectively[16].

To better understand and quantify the effect of electronic energy loss on irradiation damage from ballistic collisions, different pre-damage profiles were created in the samples. Silicon ions with an energy of 900 keV produce a damage profile that peaks at ~650 nm from the sample surface. Ion fluences of 4.4, 6.3, and 12 ions nm^{-2} were chosen to produce pre-damaged states with measured fractional disorder levels of 0.36, 0.72, and close to 1.0 (a fully amorphous state is 1.0) at the damage peak, respectively[17,18]. Such profiles are deep enough to avoid any surface effects on defect dynamics (such as defects migrate to and anneal at the surface) and readily accessible for detailed microstructural analysis and ion channeling measurements with good depth resolution.

Ionization-induced defect annealing studies were performed at room temperature using a range of ions (C, O, Si, and Ni) with energies varying from 4.5 to 21 MeV. The ion flux was 1.7×10^{12} $cm^{-2}s^{-1}$ for C and O,

1.7×10^{11} $cm^{-2}s^{-1}$ for Si, and 1.5×10^{11} $cm^{-2}s^{-1}$ for Ni, respectively. Possible bulk temperature increase over the irradiated depth due to beam heating is less than 10 degree. The corresponding stopping powers of the MeV ions are shown in Supplementary Fig. 1. Under such conditions, the electronic energy loss ranges from ~1.6 to 8.2 keV nm^{-1} at the surface and from 1.9 to 7.2 keV nm^{-1} at the damage peak region. The ratio of electronic to nuclear energy loss ranges from ~120 to 500 at the surface and from 84 to 395 at a depth of 650 nm. Moreover, the nuclear stopping is negligible, as shown in the insets (Supplementary Fig. 1), being less than 0.1 keV nm^{-1} for the Ni ions, ~0.01 keV nm^{-1} for the Si ions, and much less than 0.01 keV nm^{-1} for the O and C ions. Study of the ionization-induced recovery using these ions and energies with high electronic-to-nuclear ratios allows investigation of the separate responses of the pre-damaged materials to electronic energy deposition. As shown in Supplementary Fig. 1, after an ion fluence of 1 ion nm^{-2}, the average energy deposited to electrons at the pre-damaged peak is 19, 27, 52, and 75 eV $atom^{-1}$ for C, O, Si, and Ni, respectively. The corresponding energy deposited to the atomic nuclei is much less, only 0.05, 0.08, 0.12, and 0.9 eV $atom^{-1}$ for C, O, Si, and Ni, respectively. The MeV irradiation conditions were chosen to minimize the effect of additional cascade damage and to quantify the effect of electronic energy deposition.

Ion Channeling Measurements

The ion channeling spectra along the <0001> direction of the undamaged (virgin) SiC and its response under 21 MeV Ni irradiation are shown in Supplementary Fig. 2, and the spectra of the pre-damaged samples before and after 21 MeV Ni irradiation are shown in Supplementary Fig. 3. In these ion channeling measurements, 3.5 MeV He with a scattering angle of 155° was chosen, so that the interaction between He ions from C atoms is above the Coulomb barrier and non-Rutherford backscattering (NRBS) analysis becomes possible. Compared with normal Rutherford backscattering spectrometry (RBS) using 2.0 MeV He, the much higher non-Rutherford cross sections lead to an enhanced detection efficiency of displaced C atoms in the sublattice[19]. While 3.5 MeV He ions still undergo conventional RBS with the displaced Si atoms, this combined RBS and NRBS apparatus enables quantification of disorder on both the Si and C sublattices from a single channeling backscattering measurement. This is clearly evident by the large step height at channels of ~460 and ~1030 (marked by arrows in Supplementary Fig. 2) that correspond to the He ions backscattered from the sample surface by C and Si atoms, respectively.

The backscattering spectra from the virgin SiC and Ni-irradiated samples to ion fluences of 1, 5, and 10 ions nm^{-2} are shown in Supplementary Fig. 2a, together with a detailed comparison of backscattering yield on the Si sublattice shown in Supplementary Fig. 2b. Given the low nuclear stopping values (Supplementary Fig. 1) and high electronic-to-nuclear energy loss ratio of the 21 MeV Ni ions, negligible damage buildup within the first micrometer of the surface region is expected. The results in Supplementary Fig. 2 confirm that no visible damage is detected in the near-surface region on either the Si or C sublattice under 21 MeV Ni irradiation with ion fluences of up to 10 ions nm^{-2} (Supplementary Fig. 2b). The narrow surface peaks and the low minimum yield, χ_{min}, of less than 1.5% right after the surface peak (Supplementary Fig. 2b), suggest a high crystalline quality. Similar ion channeling measurements were conducted for the C, O, and Si irradiated samples, and negligible damage buildup within the first micrometer is confirmed with an ion fluence of up to 40 ions nm^{-2} for 4.5 MeV C and 6.5 MeV O irradiation, and up to 10 ions nm^{-2} for 21 MeV Si irradiation.

Significant ionization-induced damage recovery is observed under the MeV ion irradiation. As shown by the channeling spectrum in Supplementary Fig. 3, high disorder on both the Si and C sublattices (marked by arrows at the Si and C pre-damaged peaks) is observed after the initial 900 keV Si irradiation to a fluence of 6.3 ions nm^{-2}. Clear damage reduction on both the Si and C sublattices is observed after 21 MeV Ni irradiation over the entire damage profile. For the lowest irradiation fluence of 0.2 ion nm^{-2}, the average electronic energy deposition is ~15 eV $atom^{-1}$ (Supplementary Fig. 1); and damage reduction is clearly evident, as indicated by the decreased backscattered yield in the damage peak region (channel ~220 and ~825). Considerable damage recovery is observed with Ni irradiation at fluences up to 1 ion

nm^{-2}. After irradiation to an ion fluence of 10 ions nm^{-2} (~750 eV $atom^{-1}$), the pre-damaged state is nearly completely healed. The results in Supplementary Fig. 3 indicate that energy deposited to the target electron system can effectively anneal pre-existing damage.

Molecular Dynamics Simulations

The molecular dynamics (MD) code PARCAS[20] was used to simulate the effect of electronic energy loss on pre-damaged defective states in SiC. The Gao-Weber potential[21] was used to describe the Si–C interactions. The Gao-Weber potential[21], originally developed for 3C-SiC, considers only short range interactions with a cut-off between the first and second nearest neighbor shells. Since both 3C and 4H SiC are characterized by four-fold coordination, the only difference occurs in the second and outer neighbor shells. Polytypism does not have a strong impact on more compact and isotropic defects (vacancies, antisites, hexagonal interstitials, and many dumbbell interstitials); however, the structure and energetics of some complex and anisotropic dumbbells exhibit more dependence on polytype[22]. Consequently, the accuracy of the Gao-Weber potential for 4H-SiC is expected to be similar to that for 3C-SiC, as discussed in detail by Posselt et al.[23]. The Gao-Weber potential has previously been successfully used to model defect annihilation[24], recrystallization of nano-sized amorphous structures[25], and recovery due to swift heavy ions[13,26]. The pre-damaged defective state was produced by introducing Frenkel pairs in a MD simulation cell, and most of the simulations were carried out in defective cells containing 0.1 % or 1.0% Frenkel pairs.

With the advances in available experimental and computational tools[11,12], ionization effects and the coupling of electronic and atomic processes from irradiation with energetic charged particles have become better understood over the past few years[11]. The dominant energy loss deposited into the electronic subsystem leads to highly excited electrons along the ion track, which will rapidly distribute their energy through electron–electron interactions (<0.5 ps), and subsequently transfer their energy via electron–phonon coupling to the atomic subsystem (0.1–10 ps). The most widely used method to describe this energy dissipation and transfer process is based on the two-temperature approach[11,13,14] that treats both subsystems as a coupled continuous media. The model is described mathematically by two coupled differential equations in a cylindrical geometry with heat diffusion in time (T) and space (r). The energy exchange or heat transport between the electrons and atoms are proportional to the local temperature difference and the electron–phonon coupling constant.

$$C_e(T_e)\frac{\partial T_e}{\partial t} = \frac{1}{r}\frac{\partial}{\partial r}\left[rK_e(T_e)\frac{\partial T_e}{\partial r}\right] - g(T_e - T_l) + Q_{abs} \quad (1)$$

$$C_l(T_l)\frac{\partial T_l}{\partial t} = \frac{1}{r}\frac{\partial}{\partial r}\left[rK_l(T_l)\frac{\partial T_l}{\partial r}\right] + g(T_e - T_l) \quad (2)$$

To simulate the local heating from the electronic energy loss, the radial distribution of the atomic temperature was estimated from the inelastic thermal spike model derived from solutions of the two-temperature model[14]. In the two-temperature model, a heat equation for the energy deposited to electrons, Q_{abs}, is coupled to a heat equation for the atomic structure through the electron-phonon coupling coefficient, $g(T_e - T_l)$. In the current simulations, previously determined parameters for the two-temperature model[13] were used to determine the energy deposition profiles for the thermal spike from each ion. Lattice specific heat, $C_l(T_l) = 925.65 + 0.3772 \times T_l - 7.9259\times10^{-5} \times T_l^2 - 3.1946\times10^{-7} \times T_l^{-2}$ J kg^{-1} K^{-1}, and lattice thermal conductivity, $K_l(T_l) = 1.0 / (-0.0003 + 1.05\times10^{-5} \times T_l)$ W m^{-1} K^{-1}, are dependent on the local lattice temperature T_l (in Kelvins) and taken from ref. [6]. For the electronic subsystem, Q_{abs} is the energy absorbed by the electronic system, from a passing ion, as a function of radius and time. The electronic temperature (T_e) dependent values of $C_e(T_e)$ and $K_e(T_e)$ for insulators are used[27], modified for the 4H-SiC band gap of 3.26 eV[28]. They have constant values for T_e higher than 27000 K (from the band

gap of 3.26 eV), and drop linearly to zero with the local electronic temperature T_e at lower temperatures: $C_e = 1.0 - 3.7037\times10^{-5} \times (27\times10^3\,K - T_e)$ J m^{-3} K^{-1} for $T_e < 27000$ K, and 1.0 J cm^{-3} K^{-1} for $T_e > 27000$ K; $K_e = 2.0 - 7.4074\times10^{-5} \times (27\times10^3 - T_e)$ W cm^{-1} K^{-1} for $T_e < 27000$ K, and 2.0 W cm^{-1} K^{-1} for $T_e > 27000$ K. The electron-phonon coupling term is calculated dynamically as $g = K_e / \lambda^2$, as described elsewhere[27], where $\lambda = 5.6$ nm is the electron mean free path for SiC[13]. The initial excitation of the electronic system, Q_{abs}, is obtained[27,29], utilizing 9.29 V as the 1st ionization potential and electronic stopping values of 7.2 keV nm^{-1} (16 MeV Ni ions) and 2.6 keV nm^{-1} (6.5 MeV O ions) from SRIM[15]. For a given initial energy distribution in the electronic system from the electronic energy loss of the ions, these coupled two-temperature model equations are solved to estimate the local temperature from ionization as a function of time and radial distance from the ion trajectory[11], as shown in Supplementary Fig. 4 for the electronic energy loss from 16 MeV Ni ions in the system containing 0.1% defects, and the maximum temperature profile is used to determine the radial energy deposition profile to the lattice in the inelastic thermal spike model. While high density of defects may modify energy deposition to the cell due to a decrease in thermal conductivity and electron mobility[11], to demonstrate the direct effect of the defect density, the same energy deposition of crystalline SiC was used for both defect densities. These energy deposition profiles for the pre-damaged system were used as input in the MD simulations of recovery from 16 MeV Ni and 6.5 MeV O ions along the <0001> direction that are shown in Fig. 3 of the main text. Thermally controlled 0.5 nm thick boundary layers at the x and y boundaries of the cubic 24×24×24 nm^3 MD cell mimicked heat transfer outside the cell, and the cell was cooled to 300 K in 120 ps between each directly overlapping ion.

In our MD simulations, ions (represented as energy deposition profiles from the electronic excitation only) directly overlap, meaning the exact same location and direction where the ions hit the simulation cell. The initial damage state is marked as (0), and the number of overlapped ions "1" is the first ion. The coordination defects are calculated within an 8 nm radius cylinder around the ion path at the end of each 120 ps MD calculation. It is worth noting that it is more important to compare the trends of reduction of surviving defects with increasing events of ion impact, as shown in Figs. 2 (experiments) and 3 (simulations). Due to the direct overlap, using the MD effective radius (8 nm) to compare to experimental fluence is somewhat arbitrary. One could randomize the ion path within the MD simulation to imitate the experiment condition and to obtain a rough correlation with ion fluence based on relative healing rates, but this more costly approach will not affect the trend observed in Figs. 2 and 3 or provide different insights on ordered/disordered atomic motion for multiple ion events.

Determination of Atomic Displacements

The contrast in high-angle annular dark field (HAADF) images is sensitive to the electron channeling in a material oriented along a specific zone axis[30]. Since electron channeling can be disturbed by any lattice distortion and various defects, and is evident in HAADF images in terms of displacements in atomic positions and contrast, the quantified atomic displacements can be used as parameters for quantitative analysis of the degree of damage in crystalline materials[31]. A representative HAADF image is used to describe the atomic displacement analysis (Supplementary Fig. 5). As shown in the HAADF image, the center of each atom position is determined and is indicated by a pink marker in the image. A model of the Si sublattice in 4H-SiC is shown with an atomic arrangement in which atoms are slightly displaced (marked as Δx and Δy) from their original ideal positions. The vector modulus of the Si atom displacement is, therefore, determined as $\sqrt{\Delta x^2 + \Delta y^2}$.

Determination of Scattering Cross Section

For a single active recovery cross section, σ, the relative disorder, S, should decrease exponentially with ion fluence, Φ, as given by the expression:

$$S = S_0 - S_r\,(1 - \exp(-\sigma\Phi)), \qquad (3)$$

where S_0 is the initial disorder level and S_r is the effective recoverable level of disorder associated with the recovery cross section. Equation 3 was used to fit the data in Fig. 2b of the main text for 4.5 MeV C ions and to determine the recovery cross section shown in Fig. 5 of the main text.

The recovery of disorder for the other ions is more accurately described by two active recovery cross sections, σ_1 and σ_2, and expressed as:

$$S = S_0 - S_{r1} (1 - \exp(-\sigma_1 \Phi)) - S_{r2} (1 - \exp(-\sigma_2 \Phi)), \quad (4)$$

where S_{r1} and S_{r2} are now the recoverable levels of disorder associated with the recovery cross sections. Equation 4 was used to fit the data with initial disorder levels of 0.36 and 0.72 for 6.5 MeV O, 21 MeV Si, and 21 MeV Ni ions, as shown in Figs. 2a and 2b of the main text, respectively. The corresponding recovery cross sections are shown in Fig. 5 of the main text. Because of the complex defect structures associated with the initial disorder, both the cross sections and the recoverable disorder levels increase with increasing electronic stopping powers. In all cases, σ_1 is the dominant cross section for each ion shown in Fig. 5 of the main text, and it is about an order of magnitude larger than σ_2. The σ_2 cross section accounts for the small amount of continuous recovery at high ion fluences. Additional modeling of more complex defect structures will be required to understand the nature of these weaker recovery processes.

Supplementary References

1. Koehl, W. F., Buckley, B. B., Heremans, F. J., Calusine, G. & Awschalom, D. D. Room temperature coherent control of defect spin qubits in silicon carbide. *Nature* **479,** 84–87 (2011).
2. Smart, A. G. Silicon carbide defects hold promise for device-friendly qubits, *Phys. Today* **65,** 10–11 (2012).
3. Hristu, R., Stanciu, S. G., Tranca, D. E., Matei, A. & Stanciu, G. A. Nonlinear optical imaging of defects in cubic silicon carbide epilayers. *Sci. Rep.* **4,** 5258 (2014).
4. Castelletto, S. *et al.* A silicon carbide room-temperature single-photon source. *Nat. Mater.* **13,** 151–156 (2014).
5. Snead, L. L. *et al.* Stability of SiC-matrix microencapsulated fuel constituents at relevant LWR conditions. *J. Nucl. Mater.* **448,** 389–398 (2014).
6. Snead, L. L. *et al.* A. Handbook of SiC properties for fuel performance modeling. *J. Nucl. Mater.* **371,** 329–377 (2007).
7. Zinkle, S. J. & Busby, J. T. Structural materials for fission and fusion energy. *Mater. Today.* **12,** 12–19 (2009).
8. International Atomic Energy Agency. Viability of inert matrix fuel in reducing plutonium amounts in reactors. IAEA-TECDOC-1516, ISBN 92–0–110506–1 ISSN 1011–4289. IAEA, Vienna, Austria (2006). Printed by the IAEA in Austria, August 2006.
9. Somiya, S. Editor-in-Chief, *Handbook of Advanced Ceramics: Materials, Applications, Processing, and Properties*. Second Edition, Elsevier, ISBN: 978-0-12-385469-8 (2013).
10. Light Water Reactor Sustainability Program–*Advanced LWR Nuclear Fuel Cladding System Development: Technical Program Plan*. INL/MIS-12-25696, US Department of Energy, Office of Nuclear Energy, December (2012).
11. Weber, W. J., Duffy, D. M., Thomé, L. & Zhang, Y. The role of electronic energy loss in ion beam modification of materials. *Curr. Opin. Solid State Mater. Sci.* **19,** 1–11 (2015).

12. Zhang, Y., Debelle, A., Boulle, A., Kluth, P. & Tuomisto, F. Advanced techniques for characterization of ion beam modified materials. *Curr. Opin. Solid State Mater. Sci.* **19,** 19–28 (2015).
13. Backman, M. *et al.* Molecular dynamics simulations of swift heavy ion induced defect recovery in SiC. *Comput. Mater. Sci.* **67,** 261–265 (2013).
14. Toulemonde, M. *et al.* Experimental phenomena and thermal spike model description of ion tracks in amorphisable inorganic insulators. *Mat. Fys. Medd. Dan. Vid. Selsk.* **52,** 263 (2006).
15. Ziegler, F. J., Biersack J. P. & Ziegler, M. D. *SRIM–The Stopping and Range of Ions in Solids*, SRIM Co., Chester, MD, 2008. *The Stopping and Range of Ions in Solids*. Ziegler, J. F., Biersack J. P. & Littmark, U. Pergamon, New York, 1985.
16. Zhang, Y. *et al.* Effects of implantation temperature on damage accumulation in Al-implanted 4H-SiC. *J. Appl. Phys.* **95,** 4012–4018 (2004).
17. Zhang, Y. *et al.* Damage Accumulation and Defect Relaxation in 4H Silicon-Carbide. *Phys. Rev. B* **70,** 125203 (2004).
18. Zhang, Y. *et al.* Damage Evolution on Sm and O Sublattices in Au-implanted Samarium Titanate Pyrochlore. *J. Appl. Phys.* **95,** 2866–2872 (2004).
19. Zhang, Y. *et al.* New ion beam materials laboratory for materials modification and irradiation effects research. *Nucl. Instrum. Methods B* **338,** 19–30 (2014).
20. Nordlund, K. *et al.* Defect production in collision cascades in elemental semiconductors and fcc metals. *Phys. Rev. B* **57,** 7556–7570 (1998).
21. Gao, F. & Weber, W. J. Empirical potential, defect formation and properties in SiC, *Nucl. Instrum. Methods B* **191,** 504–508 (2002).
22. Posselt, M., Gao, F. & Weber, W. J. Atomistic simulations on the thermal stability of the antisite pair in 3C- and 4H-SiC. *Phys. Rev. B* **73,** 125206 (2006).
23. Posselt, M., Gao, F., Weber, W. J. & Belko, V. A comparative study of the structure and energetics of elementary defects in 3C- and 4H-SiC. *J. Phys. Cond. Matter* **16,** 1307–1323 (2004).
24. Gao, F. & Weber, W. J. Recovery of close Frenkel pairs produced by low energy recoils in SiC. *J. Appl. Phys.* **94**, 4348–4356 (2003).
25. Gao, F., Devanathan, R., Zhang, Y. & Weber, W. J. Annealing simulations of nano-sized amorphous structures in SiC. *Nucl. Instrum. Methods B* **228**, 282–287 (2005).
26. Debelle, A. *et al.* Combined experimental and computational study of the recrystallization process induced by electronic interactions of swift heavy ions with silicon carbide crystals. *Phys. Rev. B* **86**, 100102 (2012).
27. Dufour, Ch., Khomenkov, V., Rizza, G. & Toulemonde, M. Ion-matter interaction: the three-dimensional version of the thermal spike model. Application to nanoparticle irradiation with swift heavy ions. *J. Phys. D: Appl. Phys.* **45,** 065302 (2012).
28. Goldberg Yu., Levinshtein M. E. & Rumyantsev S. L. Properties of Advanced Semiconductor Materials GaN, AlN, SiC, BN, SiC, SiGe. Eds. Levinshtein, M. E., Rumyantsev, S. L. & Shur, M. S. John Wiley & Sons, Inc., New York, ISBN: 978-0-471-35827-5, 93-148, 2001.
29. Waligórski, R. P. M., Hamm, R. N. & Katz, R. The Radial Distribution of Dose around the Path of a Heavy Ion in Liquid Water. *Nucl. Tracks Meas.* **11,** 309 (1986).
30. Yu, Z., Muller, D. A. & Silcox, J. Effects of specimen tilt in ADF-STEM imaging of a-Si/c-Si interfaces. *Ultramicroscopy* **108,** 494–501 (2008).
31. Kim, Y.-M. *et al.* Interplay of octahedral tilts and polar order in $BiFeO_3$ films. *Adv. Mater.* **25,** 2497–2504 (2013).
